# Characteristics and function of the gut microbiota in patients with rectal neuroendocrine tumors

**DOI:** 10.7150/jca.103297

**Published:** 2025-01-01

**Authors:** Yue Gao, Hongxia Zheng, Mujie Ye, Guozhi Zhou, Jinhao Chen, Xinyun Qiang, Jianan Bai, Xintong Lu, Qiyun Tang

**Affiliations:** Department of Geriatric Gastroenterology, Neuroendocrine Tumor Center, The First Affiliated Hospital of Nanjing Medical University, No. 300 Guangzhou Road, Nanjing 210029, Jiangsu Province, China.

**Keywords:** rectal neuroendocrine tumors, gut microbiome, liver metastasis, metagenomic sequencing

## Abstract

The gut microbiota plays a significant role in the initiation and progression of tumors, but its role in rectal neuroendocrine tumors (rNETs) remains unclear. Fecal samples were collected from 19 healthy individuals and 21 rNET patients,with the rNET cohort further divided into metastatic (rNETs-M) and non-metastatic (rNETs-nM) groups. Using metagenomic high-throughput sequencing, we analyzed the diversity, species composition, and functional characteristics of the gut microbiota. We applied a random forest model to identify potential microbial biomarkers for predicting rNET and specifically distinguishing rNETs-M cases. Alpha diversity analysis revealed that species diversity was lower in the rNETs group than in the control group. In contrast, the rNETs-M group exhibited higher species diversity than the rNETs-nM group. Beta diversity analysis demonstrated significant differences in community structure between the rNETs and control groups between the rNET-M and rNETs-nM groups. Notably, in the rNETs group, the abundance of potential pathogens such as Escherichia coli and Shigella was elevated.Furthermore, the rNETs-M group exhibited an increased abundance of potential pathogens such as Alistipes. KEGG enrichment analysis indicated that these distinct microbiota play a significant role in environmental information processing, genetic information processing, and metabolic pathways. Random forest analysis and ROC curve results identified Lachnospira pectinoschiza (AUC=0.885), Parasutterella muris (AUC=0.862), Sodaliphilus pleomorphus(AUC=0.956), Methylobacterium iners (AUC=0.971) as biomarkers with significant discriminatory value.

## Introduction

Neuroendocrine neoplasms (NENs) are highly heterogeneous tumors originating from neuroendocrine cells and peptidergic neurons that produce polypeptide hormones and bioactive amines 1. NENs can occur in any part of the body, with gastroenteropancreatic neuroendocrine tumors (GEP-NENs) being the most common, accounting for 55%-70% of all NENs 2. In recent years, the incidence of GEP-NENs has continued to rise, increasing from 1.09 per 100,000 in 1973 to 6.98 per 100,000 in 2012, making it the second most common digestive system cancer 3. The rectum is a frequent site for NENs, and because patients with rectal neuroendocrine tumors (rNETs) often lack obvious clinical symptoms, timely diagnosis and treatment can be challenging 4. Although the prognosis of rNETs is generally better compared to most tumors, over 20% of patients typically present with distant metastasis at diagnosis, with liver metastasis being the most common site 5. Studies have shown that patients with liver metastasis have a significantly lower five-year survival rate compared to those without liver metastasis 6.

In recent years, increasing research has shown that microbes play an important role in human health and disease, with the gut microbiota producing many essential metabolites to maintain host and gut homeostasis 7. With the advancement of metagenomic sequencing technology, studies have found that the gut microbiota can promote tumor initiation and progression through DNA mutations, activation of oncogenic pathways, promotion of chronic inflammation, supplementation of systemic pathways, and initiation of metastasis 8]-10. Research on gut microbiota in NENs is increasing, with metagenomic sequencing of fecal samples from patients with midgut NENs and healthy controls showing differences in gut microbiota composition and function, indicating a potential link between the gut microbiota and midgut NENs 11. A study found that rNETs patients exhibited abnormal depletion of microbial species and weakened connectivity, as well as abnormal accumulation of lipids and lipid-like molecules, suggesting that the occurrence of rNETs may be related to a weakened gut microbiota activity in energy metabolism, vitamin biosynthesis, and transport 12.

In this study, we used metagenomic sequencing to analyze the species-level composition of gut microbiota and their potential functions and metabolic characteristics in patients with rNETs, as well as in those with liver metastasis. The results may help identify biomarkers and potential therapeutic targets for rNETs with liver metastasis.

## Materials and methods

### Research subjects

Stool samples were obtained from 21 patients at the Neuroendocrine Tumor Center, the First Affiliated Hospital of Nanjing Medical University, and 19 healthy individuals at the physical examination center.

The inclusion criteria for the control group were as follows: (1) age >18 years old and <80 years old; (2) negative results on OC-qFIT ultra-sensitive quantitative fecal occult blood tests. The inclusion criteria for the rNETs group were as follows: (1) Pathologically diagnosed with rNETs; (2) Imaging diagnosis of liver space-occupying lesions.

Exclusion criteria are as follows: (1) Patients who had taken antibiotics, corticosteroids, or probiotics within 3 months before sample collection; (2) Patients who have undergone abdominal surgery or other invasive treatments within 3 months before sample collection; (3) Patients who had taken cathartic agents or underwent enema treatment before sample collection; (4) Personal history of cancer, family history or personal history of inflammatory bowel disease; (5) lactation or pregnancy; (6) preoperative radiotherapy or neoadjuvant chemotherapy; (7) Patients who have undergone fecal bacteria transplantation in the past; (8) Individuals with mental illness; (9) incomplete information or lack of informed consent; (10)Established or suspected history of alcohol or drug abuse.

### Fecal sample collection

In accordance with the predefined inclusion and exclusion criteria, a total of 40 fecal samples meeting the specified standards were collected and promptly preserved in a -80 ℃ refrigerator for future analysis. Before sample collection, all participating patients provided informed consent. This study has received approval from the Ethics Review Committee of the First Affiliated Hospital of Nanjing Medical University.

### Experimental methods

#### DNA extraction

Genomic DNA from the sample was extracted using the HiPure Bacterial DNA Kit (Guangzhou, China) following the manufacturer's protocol. The quality of the DNA was assessed using Qubi (Thermo Fisher Scientific, Waltham, MA) and Nanodrop (Thermo Fisher Scientific, Waltham, MA).

#### Illumina sequencing

The qualified genomic DNA was initially fragmented to 350 bp through acoustic shearing, followed by terminal repair, A-tailing, and ligation of Illumina sequencing adapters using the NEBNext® Ultra™DNA Library Prep Kit (NEB, USA). Subsequently, 300-400bp DNA fragments were amplified and enriched via PCR. The resulting PCR products were purified using the AMPure XP system (Beckman Coulter, Brea CA., USA). Sequencing libraries were evaluated with an Agilent 2100 bioanalyzer (Agilent Santa Clara CA.) and quantified using real-time PCR before sequencing on an Illumina Novaseq 6000 sequencer employing a PE 150 sequencing strategy.

### Statistical method

#### Quality assessment of intestinal flora sequencing

The raw data obtained after sequencing were filtered based on the following criteria: (1) removal of reads containing adapters; (2) elimination of reads with an N ratio exceeding 10%; (3) exclusion of low-quality reads, defined as those where the number of bases with a quality score Q≤20 accounted for more than 50% of the entire read. All samples' Q20 and Q30 values exceeded 95%, indicating high data quality suitable for subsequent sequencing.

#### Analysis of species composition

In the R language, the VennDiagram package is utilized to construct Venn diagrams illustrating common endemic species among species in different groups and to distinguish between species unique to each group, disregarding abundance and focusing solely on presence or absence. Visualizations were created for two species groups at both phylum and genus levels, while circus plots were employed to demonstrate abundance correlations between samples and species.

#### Alpha diversity analysis

Chao1, ACE, Shannon, Simpson index were calculated using Python scikit-bio package 13(version 0.5.6). Alpha index comparison between groups was calculated by Welch's t-test and Wilcoxon rank test in R project Vegan package[13. Alpha index comparison among groups was computed by Tukey's HSD test and Kruskal-Wallis H test in R project Vegan package^13^.

#### Beta diversity analysis

Bray-curtis distance matrix based on gene/taxon/function abundance was generated by R Vegan package 13. Multivariate statistical techniques including PCA (principal component analysis), PCoA (principal coordinates analysis) and NMDS (non-metric multidimensional scaling) of Bray- curtis distances were calculated using R vegan package 13 and plotted using R ggplot2 package 14. Adonis (also called Permanova) and Anosim test was calculated using R project Vegan package 13. Heatmap graph were plotted using R Pheatmap package^15^.

#### Difference analysis

##### Basic analysis

Between groups Venn analysis was performed in R project VennDiagram package 16 and upset plot was performed in R project UpSetR package 17to identify unique and common species or functions. Species/functions comparison between groups was calculated by welch's t-test and wilcoxon rank test in R project Vegan package^13^. Species/functions comparison among groups was computed by ANOVA (analysis of variance) in R project Vegan package^13^.

##### Personalized analysis

Species comparison between groups was calculated by Metastats (version 20090414) 18. Differentially enriched KEGG pathways were identified according to their reporter score from the Zscores of individual Kos (KEGG Orthologs). An absolute value of reporter score = 1.96 or higher (95% confidence according to a normal distribution) was used as a detection threshold for pathways that differed significantly in abundance 19. Biomarker features of species and functions in each group were screened by LEfSe software (version 1.0) 20. Ternary plot of species was plotted using R ggtern package 21 based on tukey HSD test using R Vegan package^13^.

## Results

### Library sequencing data and gene prediction

The sequencing yielded a total of 3,743,818,196 original reads. Following pre-processing, the dataset retained 3,700,381,650 clean reads available for analysis, averaging 92,509,541 reads per sample (Figure S1A). The average number of bases per sample before quality control was 14,039,318,235 bp; after quality control, it was reduced to an average of 13,829739561 bp. 345743489 high-quality reads were obtained following host sequence screening (Figure S1B). Subsequently, the giant hit software was utilized for assembling the effective readbeing 2724.575, with an average continuous length of each sample being 2724.575 and 2724.575 bp without significant inter-group differences (Figure S1C).

### Diversity of the intestinal microbiota

**Figure 1:**
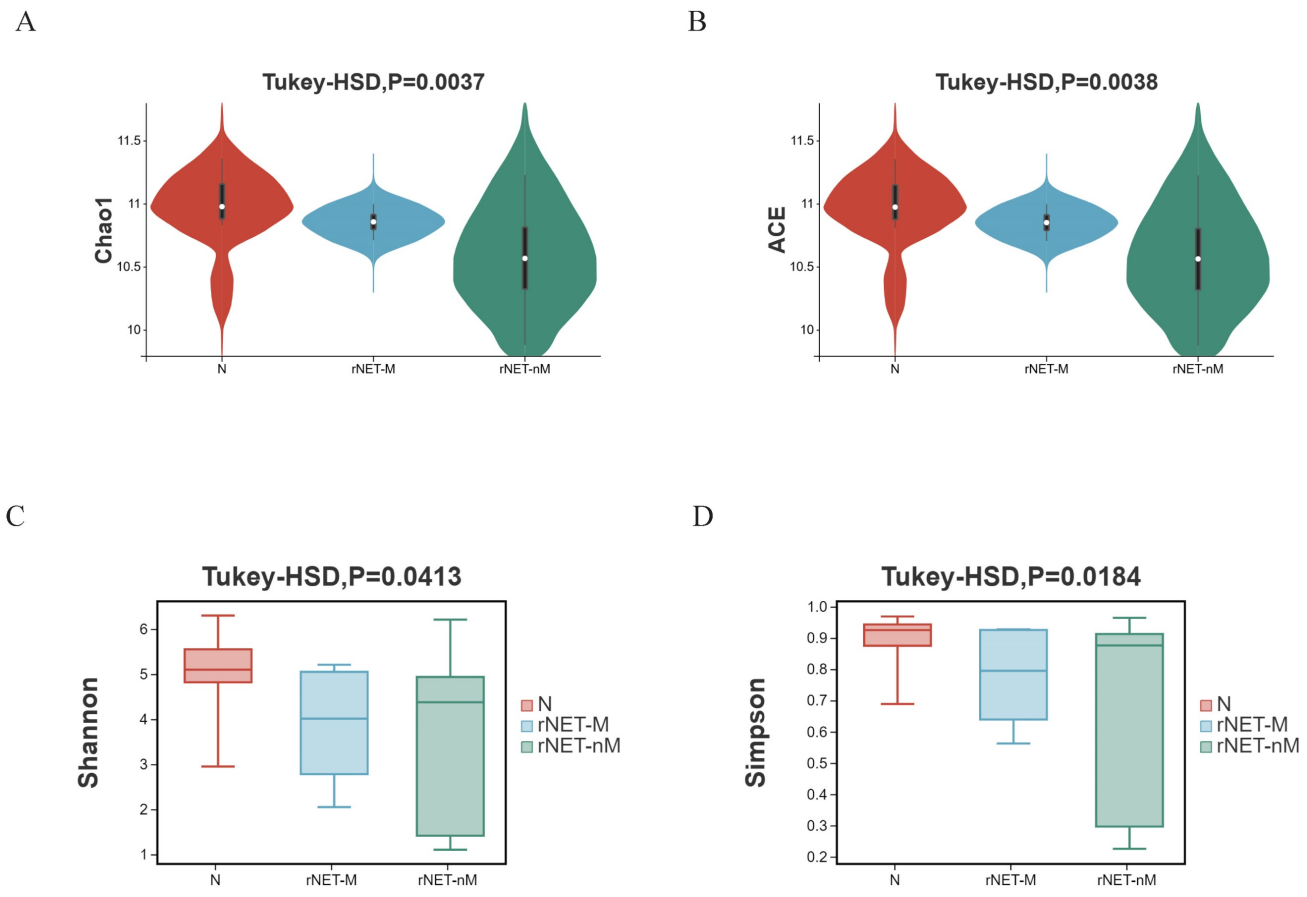
(A-D) Illustrates the alpha diversity indexes, including the Chao1 index (A), ACE index (B), Shannon index (C), and Simpson index (D) of the rNETs group, rNETs-M group, and control group.

Alpha diversity illustrates the box chart and P-values for comparing indicators: (A) Chao1 index, (B) ACE index, (C) Shannon index, and (D) Simpson index. The Chao1 and ACE indices exclusively assess species richness, with higher values indicating greater diversity. In contrast, The Shannon index and Simpson index provide a comprehensive reflection of species richness and evenness. A higher value indicates a greater sample balance. Comparisons were conducted between the rNETs group and the control group, the rNETs-M group and the rNETs-nM group, and among the rNET group, the rNETs-M group, and the control group. Statistically significant differences in the four indexes were observed between the rNETs and control groups (Fig. S2). When comparing the stool samples of patients in the rNETs-M group and rNETs-nM group, a statistically significant difference was observed in the ACE index and Chao1 index. However, no significant difference was found in the Shannon index and Simpson index (Fig. S3). Statistically significant differences were observed among the three groups for all four indexes (Fig. 1).

**Figure 2:**
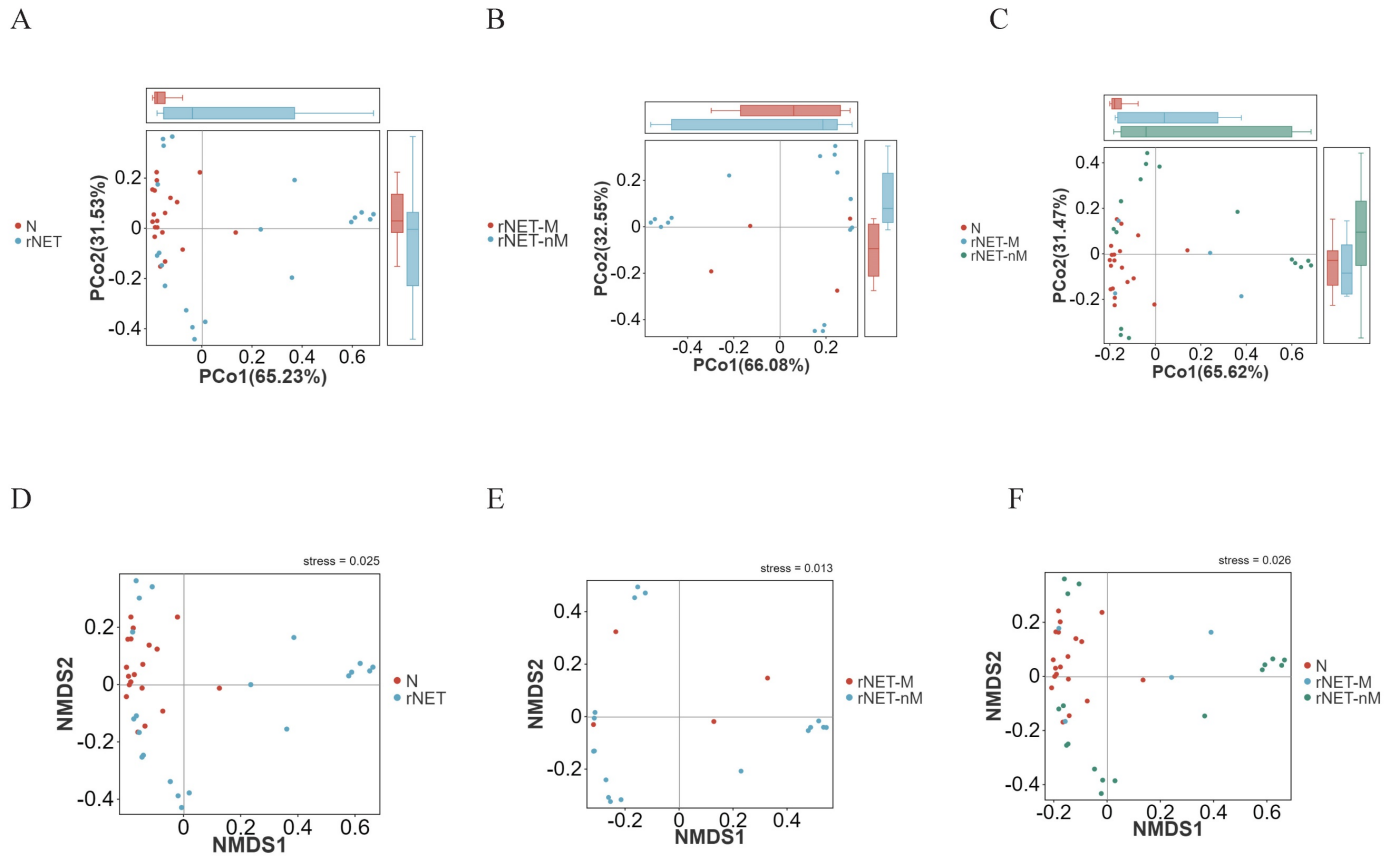
(A-C) Principal coordinates analysis (PCoA) plot depicting (A) N vs rNETs, (B) rNETs-M vs rNETs-nM, and (C) N vs rNETs-M vs rNETs-nM. (D-E) Presents the Nonmetric Multidimensional Scaling (NMDS) plot, with (D) N vs. rNETs, (E) rNETs-M vs. rNETs-nM, and (F) N vs. rNETs-M vs. rNETs-nM.

The structural similarity was explored using PCoA analysis of beta diversity. Factor 1 explained 65.23% of the total variance in both the rNETs group and control group (Fig. 2A). In comparison, Factor 2 accounted for 31.53% of the total variance. In contrast, Factor 1 explained 32.55% of the total variance in the rNETs-M group and rNETs-nM (Fig. 2B), with Factor 2 accounting for 66.08% of the total variation. The rNETs, rNETs-M, and control groups (Fig. 2C) exhibited two primary axes (factors). Specifically, Factor 1 contributed 65.62% of the total variation, whereas Factor 2 accounted for 31.47%. Subsequently, NMDS analysis was conducted based on Bray-Curtis matrix obtained from PCoA analysis. The stress values for all three NMDS analysis groups, rNETs group and control group (Fig. 2D), rNETs-M group and rNETs-nM (Fig. 2E), as well as rNETs group, rNETs-M group and control group (Fig. 2F) were all below <0.2 indicating reliable results from variance analysis.

### Characteristics of the composition of intestinal microbial species

**Figure 3:**
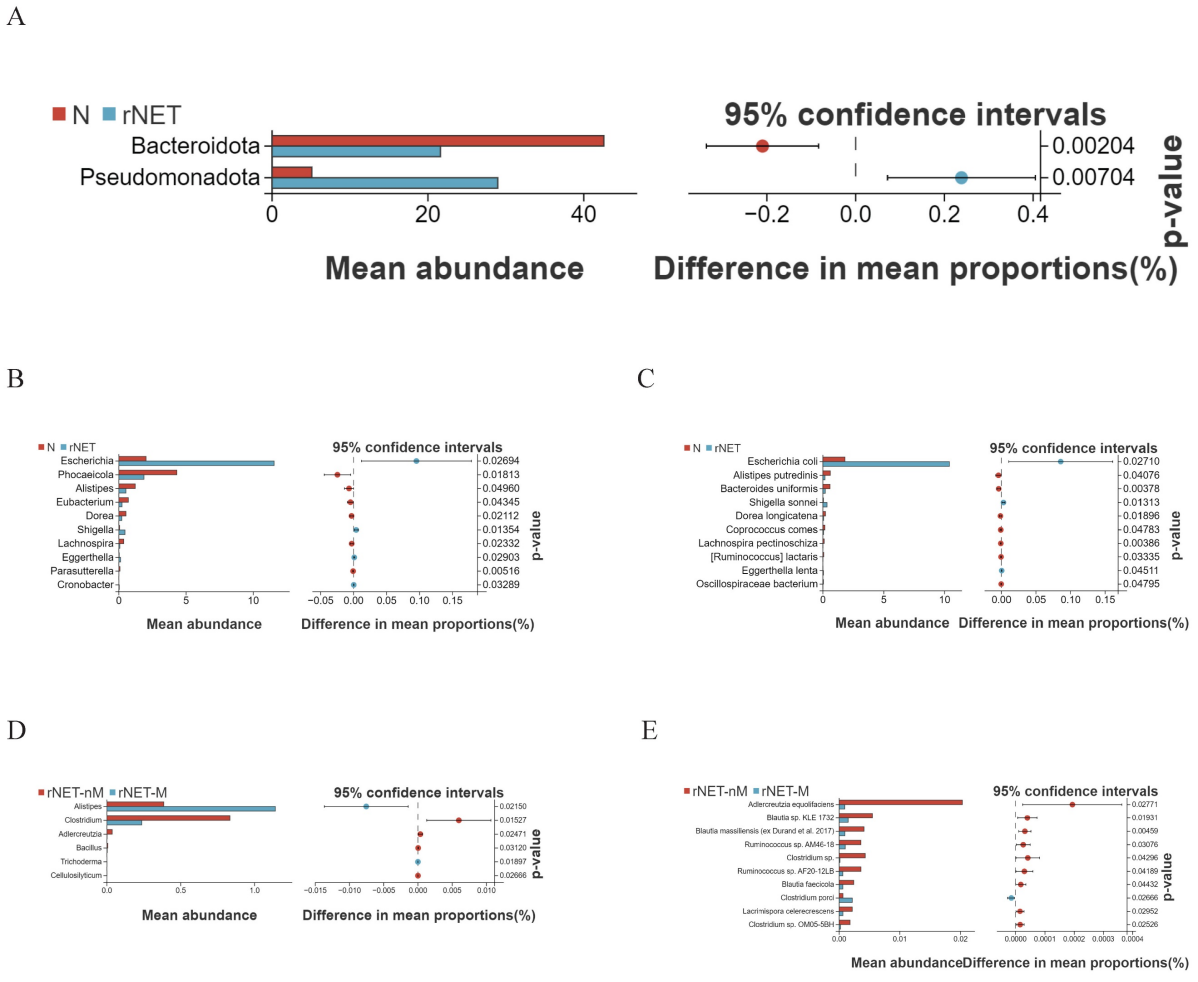
(A-E) Displays the Welch t-test results for microbial species in the intestinal microbiota of the groups N vs rNETs at the phylum levels (A), N vs rNETs at the genus levels(B), N vs rNETs at the species levels(C), rNETs-nM vs rNETs-M at the genus level (D), rNETs-nM vs rNETs-Mat the species level (E).

Welch's t-test was employed to compare the differences between the rNETs group and the control group. At the phylum level (Fig. 3A), Pseudomonadota exhibited enrichment in the tumor group. At the same time, Bacteroidota was more prevalent in the control group. At the genus level (Fig. 3B), bacteria enriched in the control group included Eubacterium, Dorea, Phocaeicola, Alistipes, and Lachnospira. Conversely, Escherichia and Shigella were present in higher abundance in the tumor group. Finally, at the species level (Fig. 3C), Alistipes putredinis, Bacteroides uniformis, and Dorea longicatena were found to be enriched in the control group. In contrast, Escherichia coli, Shigella sonnei, and Eggerthella lenta were identified as predominant strains within the tumor group.

Welch's t-test revealed distinct differences in flora between the rNETs-M and rNETs-nM groups. No statistically significant species were identified at the phylum level. At the genus level (Fig. 3D), Clostridium was found to be abundant in the rNETs-nM group, while Alistipes exhibited higher abundance in the rNETs-M group. At the species level (Fig. 3E), bacteria enriched in the rNETs-nM group included Adlercreutzia equolifaciens, Blautia faecicola, and Clostridium porci, whereas these were more prevalent in the rNETs-M group.

In the case of multiple groups, to elucidate species differences among different groups, we constructed the Wayne diagram (Fig. S4) based on species abundance information from the samples, depicting taxonomic distribution at phylum, genus, and species levels to illustrate shared endemic information across samples. At the phylum level (Fig. S4A), 62 phyla were common to all three groups, with 13 unique to the healthy group and 4 bacteria genera unique to the primary group; no specific bacteria were found in the liver metastasis group. At the genus level (Fig. S4B), 714 bacteria genera were common across all three groups, with 102 unique to the healthy group and 73 unique to the primary group. Five bacteria genera were endemic in the liver metastasis group. At the species level (Fig. S4C), there were 2773 common bacterial species; among these, 472 were endemic in the healthy group and 392 in primary tumors, while only 33 bacterial genera showed endemism in liver metastases. Trend analysis revealed no gradual increase at the phylum level but demonstrated a gradual increase at the genus level (Table 1) and species level (Table 2); these differences were statistically significant.

### Differential functional analysis of the gut microbiota

**Figure 4:**
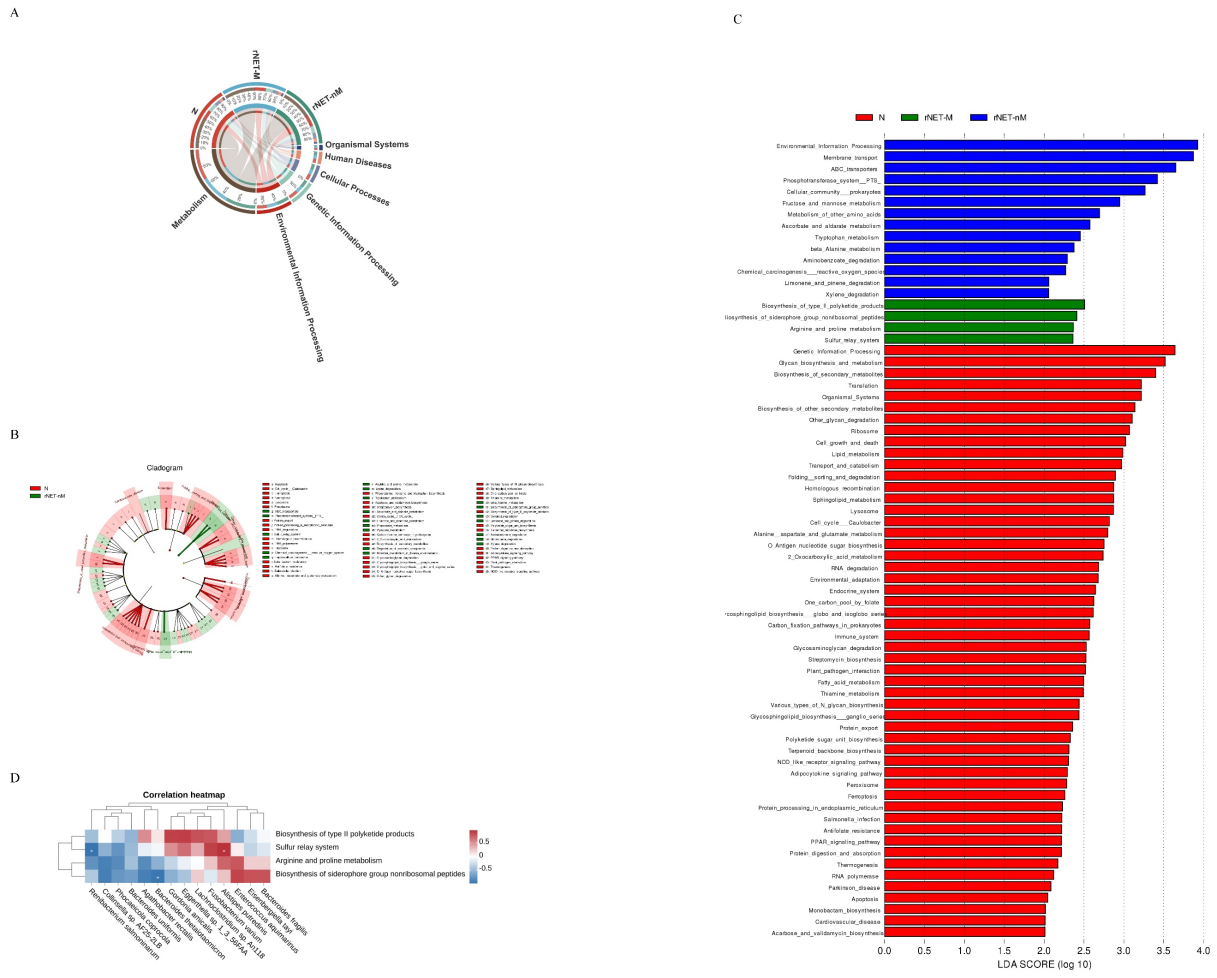
(A) Cladogram analysis of functional differences among three groups, (B) Cladogram analysis of functions among three groups, and (C) LDA score analysis of functions within the three groups. Additionally, (D) Heatmap showing the correlation between gut microbiota and metabolic functions.

In terms of exploring the functional differences of the flora, we annotated the total number of genes in all the statistical samples to the pathway database. Our analysis revealed that most of the genes were associated with metabolic functions (Fig. 4A). was depicted as a branching tree graph (Fig. 4B). LEfSe was employed to assess and compare functional differences across each group. Differential function analysis using KEGG database identified significant pathways related to Environmental Information Processing, Membrane Transport, and ABC Transporters (Fig. 4C). Furthermore, within the metastasis group, key pathways included Biosynthesis_of_type_II_polyketide_products, Biosynthesis_of_siderophore_group_nonribosomal_peptides, Arginine_and_proline_metabolism, and Sulfur_relay_system. Spearman correlation analysis (Fig. 4D) was conducted to examine associations between bacterial flora at increasing species levels and rNETs-M group bacterial flora. The results indicated a positive correlation between Alistipes putredinis and the Sulfur_relay_system pathway while Renibacterium salmoninarum showed a negative correlation with this pathway. Additionally, Bacteroides thetaiotaomicron negatively correlated with the Biosynthesis_of_siderophore_group_nonribosomal_peptides pathway.

### Random forest model for vegetation and receiver operating characteristic (ROC) analysis

**Figure 5:**
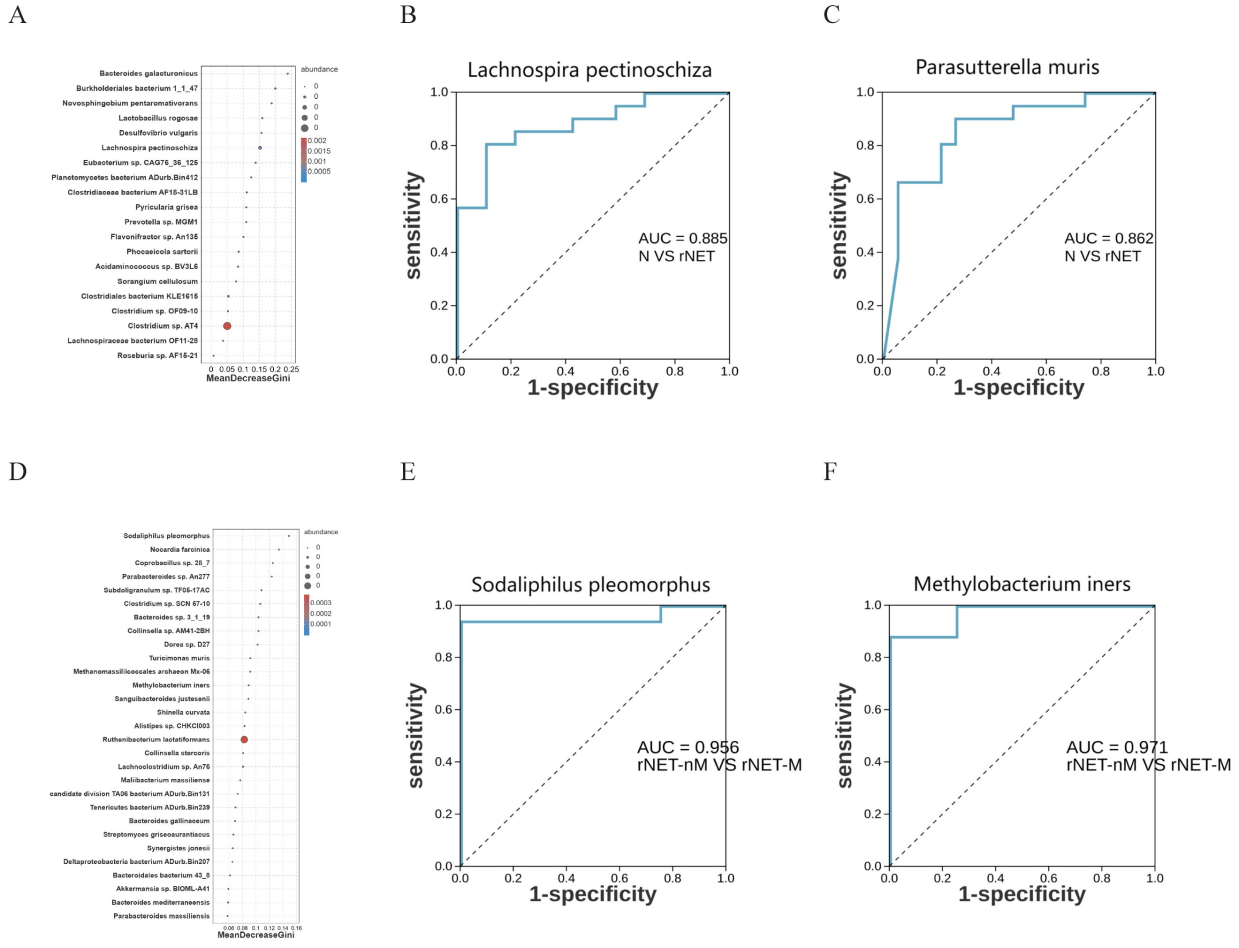
Illustrates the random forest model and ROC analysis of flora. Panel A shows the random forest model of the control, rNETs group, and groups, while panel B presents the ROC analysis of Lachnospira pectinoschiza. Additionally, panel C displays the Parasutterella ROC analysis of muris, and panel D depicts the random forest model of the rNETs-nM group and rNETs-M group. Furthermore, panels E and F showcase the ROC analyses of Sodaliphilus pleomorphic and Methylobacterium liners, respectively.

Random forest model analysis was employed to investigate the potential of microbial flora as a predictive factor for rNETs and liver metastases. To further assess the utility of intestinal microbes as biomarkers, the area under the curve (AUC) was utilized for analysis. At the species level, random forest model analysis revealed that Lachnospira pectinoschiza was pivotal in identifying rNETs (Fig. 5A). Receiver Operating Characteristic (ROC) curve analysis was conducted for different species at the species level to evaluate the sensitivity of the differential flora in diagnosing rNETs, as indicated by the area under the curve. Lachnospira pectinoschiza exhibited an AUC of 0.885 [95%CI (0.781-0.988)] (Fig. 5B).Parasutterella muris demonstrated an AUC of 0.862 [95% CI (0.744-0.981)](Fig. 5C), indicating its potential as a significant biomarker for distinguishing the rNETs group from the control group. Furthermore, the random forest analysis highlighted Sodaliphilus pleomorphus as the most influential in differentiating rNETs-M (Fig. 5D). Receiver Operating Characteristic (ROC) curve analysis was conducted at the species level, revealing that Sodaliphilus pleomorphus achieved an AUC of 0.956 [95% CI (0.865-1)] (Fig. 5E), and Methylobacterium iners attained an AUC of 0.971 [95% CI (0.901-1)] (Fig. 5F). These findings underscore their significance as crucial biomarkers for identifying the rNETs-nM group.

## Discussion

As increasing evidence suggests a link between gut microbiota dysbiosis and tumors, NENs have also garnered widespread attention. Compared to previous studies based on 16S rRNA gene sequencing, our research is the first to use metagenomic sequencing analysis to describe the gut microbiota composition at the species level in rNETs patients with liver metastasis, revealing the gene functions and potential metabolic pathways of gut microbes.

Compared to prior research 12, our study found significant differences in both α-diversity and β-diversity between the rNETs group and the control group, suggesting notable shifts in microbial community structure. Compared to healthy individuals, the richness and diversity of gut microbiota in tumor patients were significantly reduced. The rNETs-M group showed higher Chao1 and ACE indices than the rNETs-nM group, indicating increased gut microbiota diversity in patients with liver metastasis, which may be related to tumor metastasis to the liver. Song W *et al.* 22 found that, compared to primary liver cancer, the relative abundance of Fusobacterium nucleatum was significantly higher in liver metastases from colorectal cancer, and that Fusobacterium nucleatum and other symbiotic bacteria such as Bacteroides and Prevotella species colonize liver metastases as cancer cells spread. This suggests that an increase in Fusobacterium nucleatum and symbiotic bacteria may promote colorectal cancer growth and liver metastasis.

There is a dynamic balance between the microbiota and host gastrointestinal system health and disease. When microbial homeostasis is disrupted, it can lead to disease onset^23^. At the phylum level, the dominant bacterial phyla in the gut microbiota of the rNETs group and the control group are Bacteroidota and Pseudomonadota. In the rNETs group, Pseudomonadota is upregulated, while Bacteroidota is downregulated. At the genus level, gut microbiota in the rNETs group, including Escherichia and Shigella, are upregulated. At the species level, Escherichia coli, Shigella sonnei, and Eggerthella lenta are upregulated, which may be associated with various diseases. Previous research data indicate an association between inflammatory bowel disease (IBD) and intestinal NENs, with tumor risk increasing sevenfold compared to the general population 24. Studies have reported that the onset of IBD is related to the depletion of multiple beneficial bacteria, particularly the reduction of certain symbiotic bacteria, such as an imbalance in Clostridia and Firmicutes proportions and decreased Bacteroidota abundance 25. Our results suggest that Bacteroidota is downregulated in the rNETs group compared to the control group. Therefore, whether supplementing beneficial Bacteroidota can reduce the incidence of rNETs requires further investigation. Previous studies have found that Eggerthella lenta is more abundant in high-grade gastroenteropancreatic neuroendocrine tumors (GEP-NETs) than in low-grade GEP-NETs^26^, leading us to speculate that Eggerthella lenta may be closely associated with tumor development. Clinically, rNETs patients may experience non-specific symptoms such as abdominal pain and diarrhea^27^, which may be related to Shigella sonnei invading host cells, mediating multiple signaling pathways, recruiting inflammatory factors and immune cells, colonizing, and damaging colonic epithelial cells^28^]^29^. The abundance of Alistipes is elevated in the liver metastasis group; Lu Y *et al.* found that Alistipes could upregulate downstream target genes of β-catenin, activate the Wnt signaling pathway, and promote colorectal cancer metastasis^30^. Whether it can lead to liver metastasis of rNETs still needs further investigation.

To describe functional changes in the gut microbiome, we performed metagenomic functional annotation of KEGG pathways, revealing that metabolic function is the primary pathway in all three groups. LEfSe analysis showed that the 2_Oxocarboxylic_acid_metabolism pathway is upregulated in healthy individuals. Clostridium butyricum can promote the production of short-chain fatty acids (SCFAs) via the 2_Oxocarboxylic_acid_metabolism pathway, thereby repairing the gut barrier in mice with ulcerative colitis^31^. The ABC_transporters pathway is upregulated in the primary tumor group, with studies indicating that multidrug resistance (MDR) in tumor cells to chemotherapy drugs is associated with overexpression of ABC_transporters^32^. This overexpression can promote the efflux of chemotherapeutic drugs and reduce their accumulation within tumor cells^33^. Additionally, ABC_transporters can regulate the tumor immune microenvironment (TIME) by transporting various cytokines, thereby controlling antitumor immunity and sensitivity to anticancer drugs^34^. The Arginine_and_proline_metabolism pathway is significantly upregulated in the liver metastasis group, and changes in gut microbiota structure can induce intracellular toxin accumulation, leading to amino acid dysbiosis and gut barrier dysfunction by interfering with the Arginine_and_proline_metabolism pathway, potentially causing gut diseases^35^. Whether this promotes liver metastasis requires further validation.

Several consensus guidelines suggest that measuring serum CgA concentration is a reliable biomarker for identifying GEP-NENs^36^[37. However, elevated CgA levels have also been observed in some non-GEP-NENs, including lung cancer, breast cancer, and prostate cancer^38^. Previous studies have confirmed the heterogeneity of microbiome characteristics in aggressive disease groups, and an integrated analysis of microbiome markers from multiple cohorts has shown improved accuracy in early screening and diagnosis^39^. We developed a random forest model to confirm the potential of the gut microbiota in predicting rNETs. Lachnospira pectinoschiza and Parasutterella muris can serve as microbiome markers indicative of rNETs, while Sodaliphilus pleomorphus and Methylobacterium iners can be markers indicative of rNETs-M. From a clinical perspective, individuals exhibiting a "high-risk" microbiome profile may require additional colonoscopy to confirm the diagnosis.

This study has some limitations. First, all rNETs patients were from hospitals in a specific region, resulting in a limited sample size. Therefore, further validation through large-scale clinical studies across multiple regions is needed to confirm these preliminary findings. Second, our observations are primarily data-driven and only involve changes in the gut microbiota, so further *in vivo* and *in vitro* downstream validation is necessary.

## Conclusion

Metagenomic sequencing technology in this study revealed significant disparitiesin intestinal microbiota composition between patients with rNETs and the general population. Certain specific alterations in the flora maybe closely associatedwith the onset and metastasis of rNETs, offering potential insights for the diagnosis and treatment of such tumors.

## Supplementary Material

Supplementary figures.

## Figures and Tables

**Table 1:** Abundance and corresponding p-values of bacteria genera across incremental levels

Genus	N	rNETs-nM	rNETs-M	p-value	q-value
Renibacterium	0	1.00E-06	6.00E-06	0.036225282	0.360552309
Gordonia	1.20E-05	3.70E-05	7.00E-05	0.010743804	0.235198286
Oenococcus	1.10E-05	0.000105	0.000477	0.044365664	0.406185644
Agathobacter	0.0964947	0.287	0.4364	0.022394941	0.288912813
Eisenbergiella	0.003993	0.015599	0.04345	0.038245884	0.36733829
Paraprevotella	0.027287	0.05427	0.234523	0.004934687	0.235198286
Parabacteroides	0.577711	0.700713	1.615925	0.02339924	0.289841212

**Table 2:** Abundance and corresponding p-values of bacteria species across incremental levels.

Species	N	rNETs-nM	rNETs-M	p-value	q-value
Renibacterium salmoninarum	0	1.00E-06	6.00E-06	0.036225282	0.359924545
Gordonia amicalis	1.10E-05	3.20E-05	5.20E-05	0.019375022	0.283663272
Collinsella sp. AF25-2LB	3.80E-05	0.000288	0.00106	0.026768661	0.317168087
Eggerthella sp. 1_3_56FAA	8.30E-05	0.00018	0.00024	0.011361349	0.205207055
Enterococcus aquimarinus	0	4.00E-06	2.10E-05	0.039325427	0.377869186
Clostridium sp. Marseille-P3244	0.000349	0.000534	0.001267	0.037507518	0.369175123
Agathobacter rectalis	0.0964326	0.286765	0.4363	0.022386961	0.300935721
Eisenbergiella tayi	0.001754	0.004429	0.031125	0.027771232	0.324154178
Lachnoclostridium sp. An118	0.000117	0.000296	0.000776	0.010485772	0.202037667
Bacteroides fragilis	0.407747	0.840575	5.681125	0.005329296	0.202037667
Bacteroides thetaiotaomicron	0.0386905	0.208833	0.534475	0.015736526	0.258623855
Bacteroides uniformis	0.0640721	0.20402	0.242825	0.003417602	0.202037667
Phocaeicola coprocola	0.0178226	0.089142	1.172925	0.007671662	0.202037667
Alistipes putredinis	0.0668617	0.152854	0.364675	0.012966307	0.223445597
Fusobacterium varium	0.000929	0.006405	0.02401	0.007214647	0.202037667
