# Supplementary material for: Characteristics and function of the gut microbiota in patients with rectal neuroendocrine tumors
Source: J Cancer. 2025 Jan 1;16(4):1040–50. doi: 10.7150/jca.103297 (PMC11786048; doi:10.7150/jca.103297)
Supplement: Supplementary file 1 — Supplementary figures. [file jcav16p1040s1.pdf]

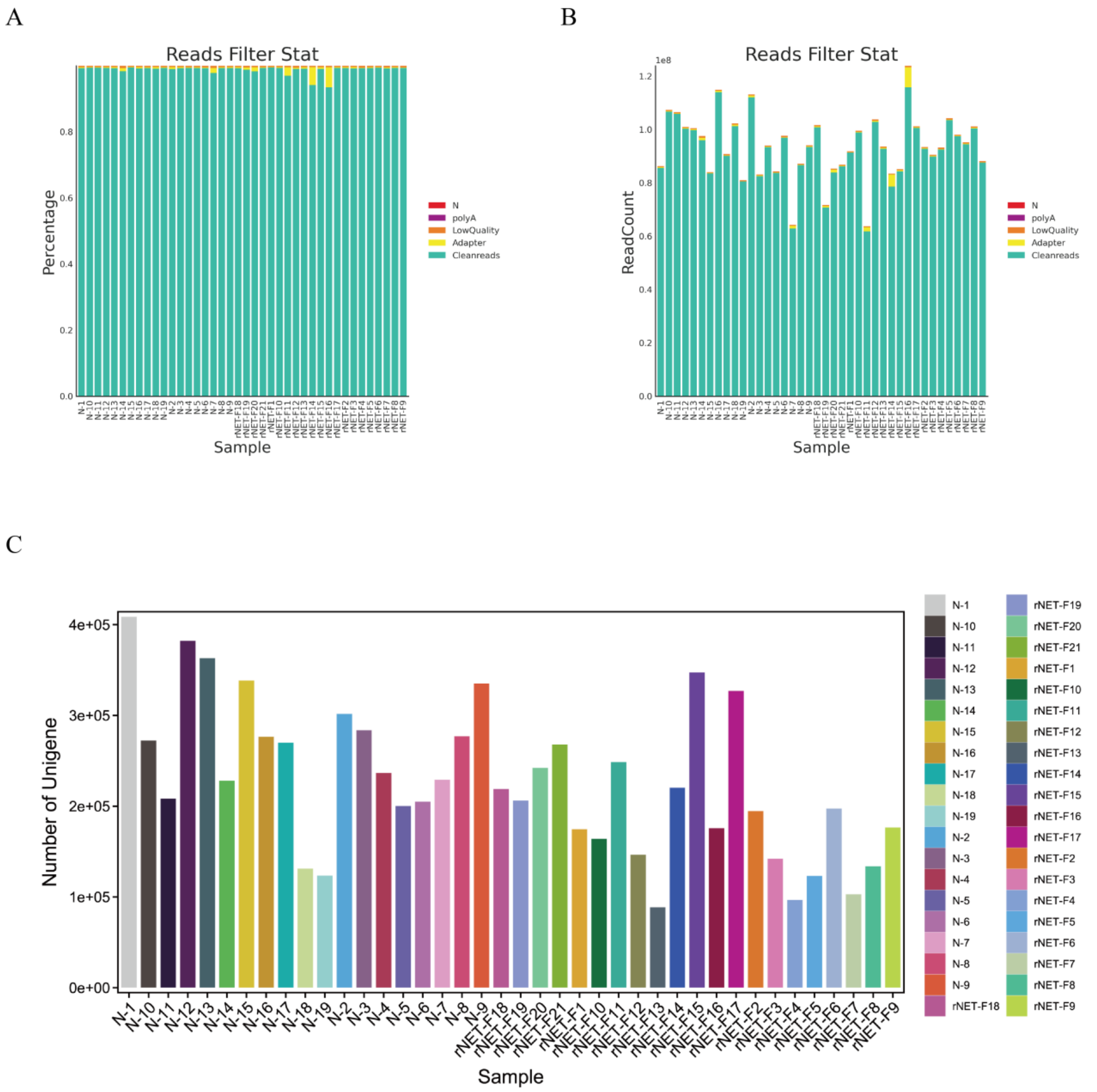

Fig. S1 (A, B) illustrates the results of raw data processing for endometrial samples (C), showing the number of unigenes.

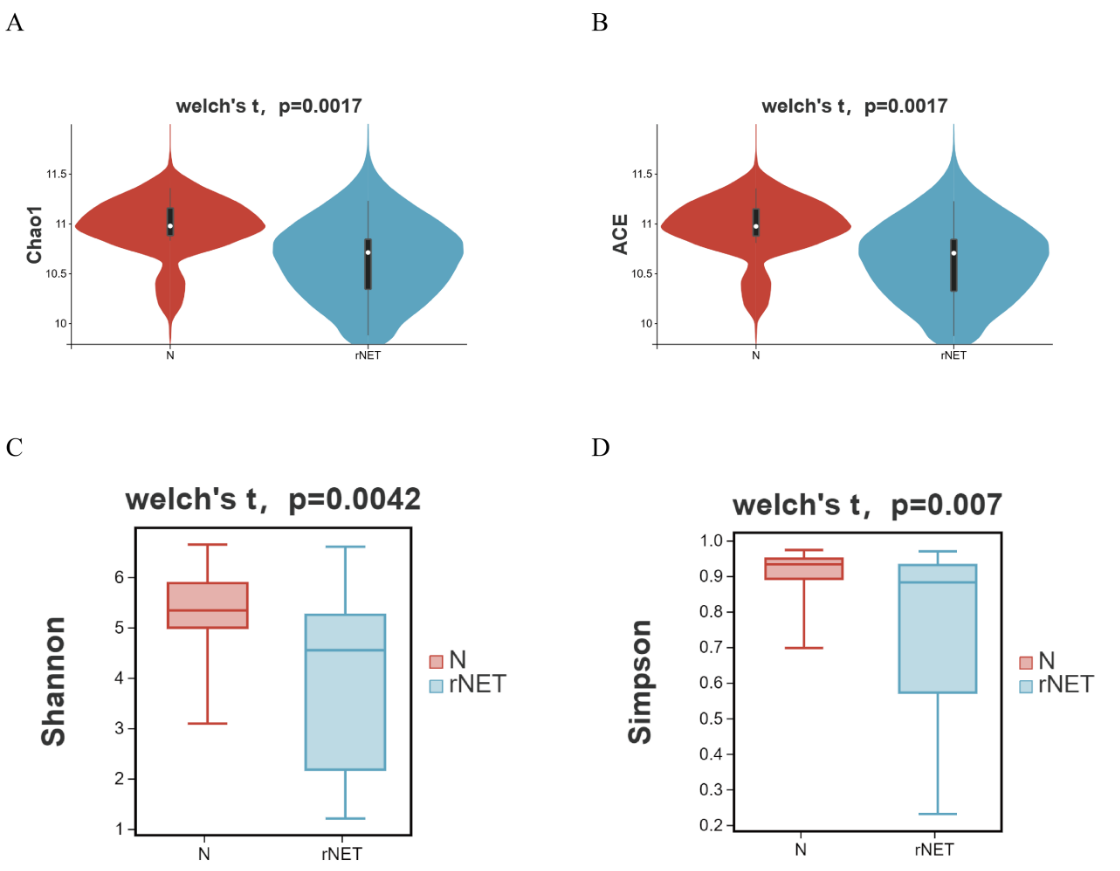

Fig S2 illustrates the comparison of alpha diversity indicators between the rNETs group and the control group, encompassing (A) Chao1 index, (B) ACE index, (C) Shannon index, and (D) Simpson index.

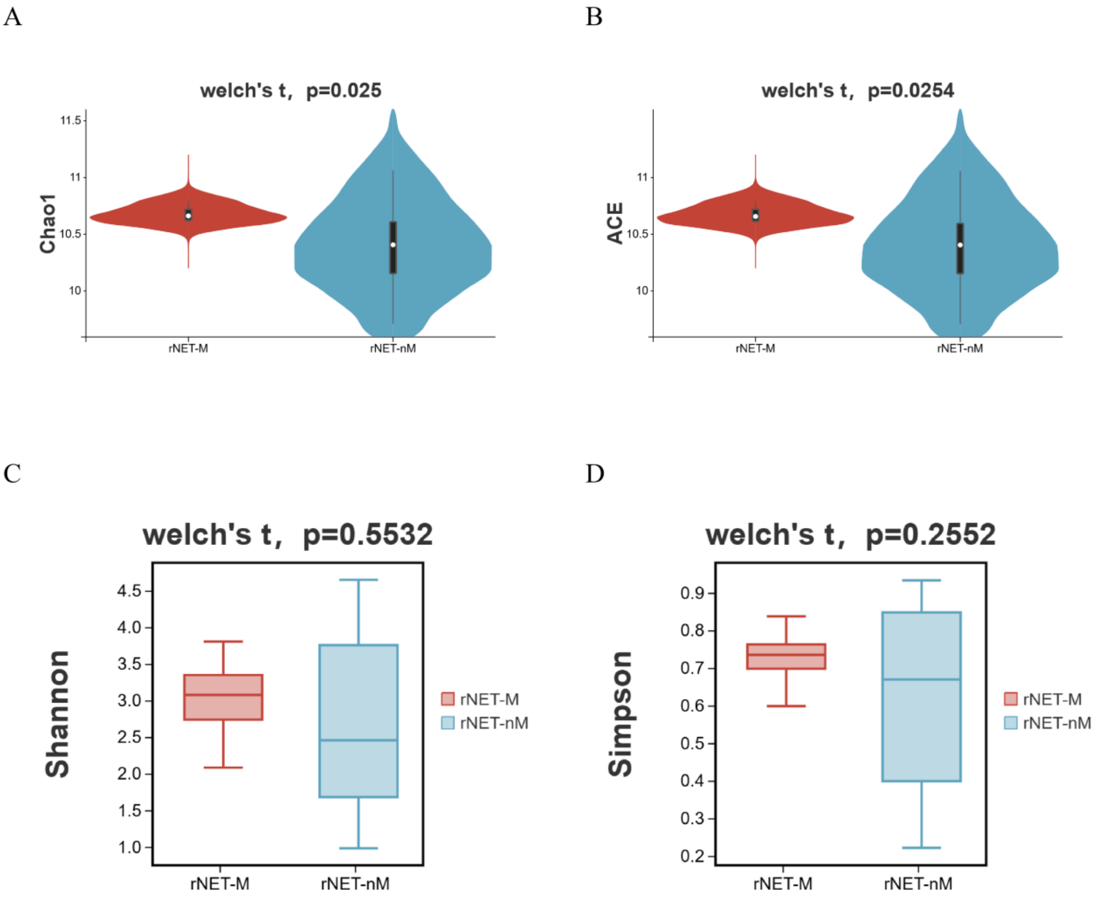

Fig. S3 presents the alpha diversity indices, including (A) the Chao1 index, (B) the ACE index, (C) the Shannon index, and (D) the Simpson index of the rNETs-M group and rNETs-nM group.

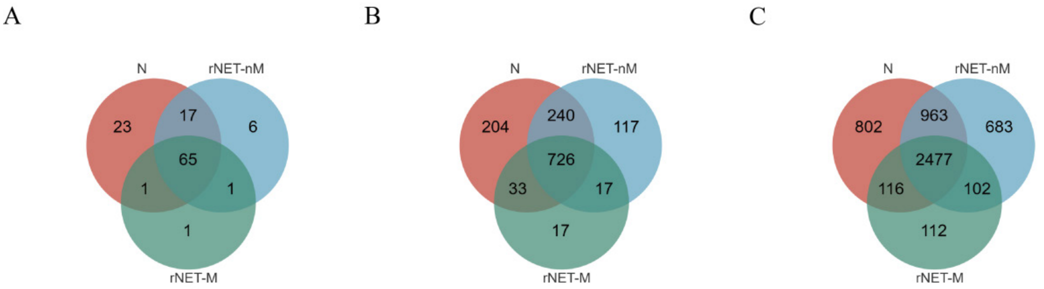

Fig. S4 N vs rNETs-nM vs rNETs-M (A) at the Phylum level, (B) at the Genus level, and (C) at the Species level.
